# Autonomic Response to the Harvard Step Test in Normal BMI and Overweight Young Adults: A Heart Rate Variability Study

**DOI:** 10.7759/cureus.111596

**Published:** 2026-06-27

**Authors:** Vishwadeepak Rajput, Vishavdeep Kaur, Ankalayya Bobbara

**Affiliations:** 1 Department of Haematology, Maharishi Markandeshwar Institute of Medical Science and Research, Mullana, Ambala, IND; 2 Department of Physiology, Maharishi Markandeshwar Institute of Medical Science and Research, Mullana, Ambala, IND

**Keywords:** autonomic function, harvard step test, heart rate variability, overweight, physical fitness index

## Abstract

Background

Excess adiposity in young adults is associated with early autonomic imbalance, preceding the development of overt cardiovascular disease. Heart rate variability (HRV) is a simple, non-invasive measure of cardiac autonomic modulation. The present study aimed to compare resting and early post-exercise HRV between normal body mass index (BMI) and overweight young adults using the Harvard Step Test.

Methodology

This comparative, analytical, observational study with repeated measures included 200 young adults aged 17-25 years, divided into a normal BMI group (n = 100) and an overweight group (n = 100). After anthropometric assessment, baseline electrocardiography (ECG) was recorded following 5 minutes of supine rest. Participants then performed the Harvard Step Test on a 23-cm stool at a rate of 40 steps per minute for a maximum of 5 minutes or until fatigue, whichever occurred earlier. Post-exercise ECG recordings were obtained for 15 minutes, and the first 5 minutes were used for early recovery HRV analysis. Time-domain HRV parameters included mean RR interval, mean heart rate (HR), standard deviation of normal-to-normal intervals (SDNN), root mean square of successive differences (RMSSD), number of pairs of adjacent normal-to-normal intervals differing by more than 50 ms (NN50 count), and percentage of NN50 counts (pNN50). Frequency-domain parameters included high-frequency (HF) power and low-frequency/high-frequency (LF/HF) ratio. The Physical Fitness Index (PFI) was also calculated. Between-group and within-group comparisons were performed, along with adjusted regression, mixed-effects modelling, and sensitivity analyses.

Results

Age and sex distribution were comparable between groups. At baseline, the overweight group demonstrated significantly lower mean RR interval, SDNN, RMSSD, NN50, and pNN50, along with higher mean HR compared with the normal BMI group (all p < 0.001). Following the exercise, both groups exhibited significant autonomic changes. However, post-exercise SDNN, RMSSD, NN50, pNN50, and HF power remained significantly lower in the overweight group. In adjusted regression analysis, overweight status was independently associated with lower baseline RMSSD (β = −20.23 ms), lower post-exercise RMSSD (β = −5.40 ms), lower post-exercise HF power (β = −82.64 ms²), and lower PFI (β = −11.36) (all p ≤ 0.001). Mixed-effects modelling demonstrated a significant group × time interaction for SDNN, RMSSD, and HF power, while mean HR did not show a significant interaction effect.

Conclusions

Overweight young adults exhibit reduced vagal-mediated HRV at rest and a less favorable early post-exercise autonomic recovery profile compared with normal BMI individuals. Among HRV indices, RMSSD and SDNN showed the most consistent alterations and may serve as useful early markers of autonomic dysfunction in this population.

## Introduction

Overweight and obesity in young adults are increasingly recognized as early contributors to cardiovascular dysregulation, with altered cardiac autonomic modulation considered a key mechanism linking excess adiposity to future cardiometabolic risk [1,2]. Heart rate variability (HRV) provides a simple, non-invasive measure of cardiac autonomic regulation, and reduced HRV in overweight individuals has been consistently associated with an adverse cardiovascular risk profile even before overt disease becomes clinically apparent [1,3].

Short-term HRV recordings are widely used for autonomic assessment due to their practicality, reproducibility, and suitability for laboratory-based physiological studies [4,5]. Among short-term HRV indices, root mean square of successive differences (RMSSD) and high-frequency (HF) components are particularly reflective of vagal (parasympathetic) activity and are commonly used to evaluate autonomic responses at rest and following physiological stress [6,7].

Previous studies in adolescents and young adults have demonstrated that increasing body mass index (BMI) is associated with reduced HRV, supporting early autonomic impairment in overweight and obese populations [2,3]. Evidence focusing on central adiposity further suggests that waist circumference and waist-to-hip ratio (WHR) may have stronger inverse associations with vagally mediated HRV. In some settings, central obesity may better discriminate autonomic dysfunction than BMI alone [8,9].

Exercise and the immediate post-exercise period provide a useful autonomic challenge, as vagal withdrawal during exertion and subsequent vagal reactivation during recovery can reveal abnormalities that may not be evident at rest [10,11]. In overweight young adults, prior studies have demonstrated higher heart rate (HR), impaired HR recovery, and delayed vagal reactivation following exercise, indicating a less favorable autonomic recovery profile compared with normal-weight individuals [10,11].

Step-based exercise tests are particularly suitable for student populations because they are simple, cost-effective, and feasible in routine physiology laboratory settings [12,13]. The Harvard Step Test, in particular, provides a practical Physical Fitness Index (PFI) and has shown associations with HRV parameters in college-aged individuals, making it appropriate for assessing both exercise tolerance and early autonomic recovery [12,13].

Medical students represent a relevant young adult cohort for such evaluation, as HRV in this population has been shown to vary with stress levels, sleep quality, and lifestyle-related factors [14,15]. However, most existing student-based studies have focused on stress, sleep, or general fitness. At the same time, comparative data on resting and early post-exercise HRV across BMI categories remain limited, particularly using a simple step-test-based protocol [13-15]. Although previous studies have demonstrated reduced HRV among overweight and obese individuals, most investigations have focused primarily on resting autonomic function. Comparative data evaluating early post-exercise autonomic recovery using a simple, low-cost, step-test-based protocol remain limited, particularly in young adults. The Harvard Step Test provides a practical physiological challenge that can reveal recovery-related autonomic abnormalities that may not be evident during resting assessment alone. Therefore, the primary objective of the present study was to compare resting and early post-exercise HRV between young adults with normal BMI and those with overweight, using the Harvard Step Test. Secondary objectives included evaluation of the PFI, assessment of autonomic recovery dynamics following exercise, and exploration of associations between central adiposity measures and vagal-mediated HRV indices.

## Materials and methods

This comparative, analytical, observational study with repeated measures was conducted in the Department of Physiology, Maharishi Markandeshwar Institute of Medical Sciences and Research, Mullana, Ambala, Haryana, India, over a period of 10 months. The study included 200 medical students aged 17-25 years, who were divided into two groups based on BMI. The normal BMI group included 100 participants with a BMI range of 18.5-24.99 kg/m², while the overweight group included 100 participants with a BMI of ≥25 kg/m².

Participants with a normal baseline electrocardiography (ECG) and written informed consent were included in the study. Individuals with hypertension, diabetes mellitus, respiratory disease, cardiovascular disease, asthma, chronic obstructive pulmonary disease (COPD), anemia, neurological or psychiatric disorders, a history of smoking or alcohol use, regular exercise habits, or any physical disability that could interfere with test performance were excluded.

Anthropometric measurements included weight, height, waist circumference, hip circumference, BMI, and WHR. BMI was calculated using the Quetelet Index, defined as body weight in kilograms divided by the square of height in meters (kg/m²), as originally described by Keys et al. [16]. The Quetelet Index is a universally accepted anthropometric calculation and does not require permission for use. After 5 minutes of supine rest, baseline ECG recordings were obtained using the Physiopac digital polygraph system. All recordings were performed in a quiet physiology laboratory under standardized conditions. Participants were instructed to avoid strenuous physical activity on the day of testing and were allowed an acclimatization period before baseline recording. ECG recordings were obtained in the supine position following 5 minutes of rest. HRV analysis was performed in accordance with the standards recommended by the Task Force of the European Society of Cardiology and the North American Society of Pacing and Electrophysiology [5], as well as with contemporary recommendations for short-term HRV assessment.

Participants then performed the Harvard Step Test according to the standardized protocol described by Brouha et al. [17]. The test was conducted on a 23-cm stool at a stepping rate of 40 steps per minute for a maximum duration of 5 minutes or until fatigue, whichever occurred earlier. The Harvard Step Test is a publicly available physical fitness assessment method and does not require permission for use. Immediately following exercise, ECG recordings were obtained for 15 minutes. For HRV analysis, only the first 5 minutes of the post-exercise recording were used, representing early recovery HRV.

HRV parameters analyzed included mean RR interval, mean HR, standard deviation of normal-to-normal intervals (SDNN), RMSSD, number of pairs of adjacent normal-to-normal intervals differing by more than 50 ms (NN50 count), percentage of NN50 counts (pNN50), HF power, and low-frequency/high-frequency (LF/HF) ratio. HRV measurements and interpretation were performed in accordance with the standards established by the Task Force of the European Society of Cardiology and the North American Society of Pacing and Electrophysiology [5], and contemporary recommendations for short-term HRV analysis [6]. The PFI, derived from the Harvard Step Test, was also analyzed. The study employed a convenience sample of eligible participants recruited consecutively during the study period. A formal a priori sample size calculation was not performed because the study was designed as an observational comparative investigation. However, a total sample of 200 participants (100 per group) was considered adequate to evaluate differences in HRV parameters between BMI categories and to support multivariable analyses.

The Physical Fitness Index (PFI) was calculated using the standard Harvard Step Test [17] formula:

\[ PFI = \frac{\text{Exercise Duration (seconds)} \times 100}{2 \times (P_1 + P_2 + P_3)} \]

Statistical analysis was performed using RStudio (R version 4.5.2, R Foundation for Statistical Computing, Vienna, Austria). Continuous variables were expressed as mean ± standard deviation, while categorical variables were presented as frequency and percentage. The chi-square test was used for categorical comparisons. An independent samples t-test was used for between-group comparisons, and a paired t-test was used for within-group pre- and post-exercise comparisons.

Linear mixed-effects models were applied for repeated-measures outcomes. Multivariable linear regression was used to assess adjusted associations for baseline RMSSD, post-exercise RMSSD, post-exercise HF power, and PFI after adjustment for age, sex, and height. Sensitivity analyses included log-transformed variables, percent change calculations, recovery ratios, and secondary analyses incorporating waist circumference and WHR. A p-value of <0.05 was considered statistically significant.

## Results

The overweight and normal BMI groups were comparable in sex distribution, with males accounting for 50 (50.0%) versus 49 (49.0%) and females accounting for 50 (50.0%) versus 51 (51.0%), respectively (p = 0.888). Mean age was also similar between groups (21.46 ± 1.95 vs 21.79 ± 2.08 years, p = 0.249). However, overweight participants had significantly higher weight, BMI, waist circumference, hip circumference, and WHR, along with lower height compared with the normal BMI group (all p < 0.001) (Table 1).

**Table 1:** Baseline demographic and anthropometric characteristics of normal BMI and overweight medical students BMI, body mass index; WHR, waist-to-hip ratio

Variable	Normal BMI (n=100)	Overweight (n=100)	p-Value
Male, n (%)	49 (49.0)	50 (50.0)	0.888
Female, n (%)	51 (51.0)	50 (50.0)	
Age (years)	21.79 ± 2.08	21.46 ± 1.95	0.249
Weight (kg)	60.72 ± 7.75	65.25 ± 10.03	<0.001
Height (cm)	168.26 ± 11.99	152.34 ± 12.28	<0.001
BMI (kg/m²)	21.45 ± 1.69	28.01 ± 1.45	<0.001
Waist circumference (cm)	78.80 ± 8.23	91.50 ± 5.61	<0.001
Hip circumference (cm)	99.59 ± 3.54	103.51 ± 2.15	<0.001
WHR	0.79 ± 0.09	0.88 ± 0.06	<0.001

At baseline, overweight participants demonstrated significantly lower mean RR interval, SDNN, RMSSD, NN50 count, and pNN50, along with higher mean HR, compared with the normal BMI group (all p < 0.001). Following exercise, the same pattern persisted, with overweight participants continuing to show significantly lower HRV indices and higher HR compared with normal BMI participants (Table 2).

**Table 2:** Baseline and early post-exercise time-domain heart rate variability indices in normal BMI and overweight medical students HRV, heart rate variability; RR, interval between consecutive R waves on electrocardiography; HR, heart rate; SDNN, standard deviation of normal-to-normal intervals; RMSSD, root mean square of successive differences; NN50, number of pairs of adjacent normal-to-normal intervals differing by more than 50 ms; pNN50, percentage of NN50 counts

Parameter	Baseline normal	Baseline overweight	p-Value	Post-exercise normal	Post-exercise overweight	p-Value
Mean RR (ms)	805.84 ± 125.76	738.29 ± 82.87	<0.001	547.56 ± 91.38	504.85 ± 86.90	0.001
Mean HR (bpm)	71.00 ± 5.83	85.35 ± 9.55	<0.001	122.23 ± 17.62	131.84 ± 16.76	<0.001
SDNN (ms)	74.74 ± 14.87	33.98 ± 9.49	<0.001	24.82 ± 8.60	15.10 ± 5.89	<0.001
RMSSD (ms)	48.06 ± 14.31	27.26 ± 7.44	<0.001	16.62 ± 8.02	11.79 ± 5.13	<0.001
NN50 (count)	100.98 ± 32.93	48.51 ± 16.73	<0.001	20.50 ± 12.52	9.82 ± 6.12	<0.001
pNN50 (%)	20.45 ± 6.02	12.76 ± 4.76	<0.001	4.93 ± 3.33	2.49 ± 1.80	<0.001

Within-group analysis demonstrated significant post-exercise autonomic changes in both groups (all p < 0.001). In both normal BMI and overweight participants, mean RR interval, SDNN, RMSSD, NN50, and pNN50 decreased significantly after exercise, while mean HR increased significantly during the early recovery period (Table 3).

**Table 3:** Pre-exercise and early post-exercise time-domain heart rate variability within normal BMI and overweight groups RR, interval between consecutive R waves on electrocardiography; HR, heart rate; SDNN, standard deviation of normal-to-normal intervals; RMSSD, root mean square of successive differences; NN50, number of pairs of adjacent normal-to-normal intervals differing by more than 50 ms; pNN50, percentage of NN50 counts

Parameter	Normal pre-exercise	Normal post-exercise	Δ Normal (post-pre)	p-Value	Overweight pre-exercise	Overweight post-exercise	Δ Overweight (post-pre)	p-Value
Mean RR (ms)	805.84 ± 125.76	547.56 ± 91.38	-258.28	<0.001	738.29 ± 82.87	504.85 ± 86.90	-233.44	<0.001
Mean HR (bpm)	71.00 ± 5.83	122.23 ± 17.62	+51.23	<0.001	85.35 ± 9.55	131.84 ± 16.76	+46.49	<0.001
SDNN (ms)	74.74 ± 14.87	24.82 ± 8.60	-49.92	<0.001	33.98 ± 9.49	15.10 ± 5.89	-18.88	<0.001
RMSSD (ms)	48.06 ± 14.31	16.62 ± 8.02	-31.44	<0.001	27.26 ± 7.44	11.79 ± 5.13	-15.47	<0.001
NN50 (count)	100.98 ± 32.93	20.50 ± 12.52	-80.48	<0.001	48.51 ± 16.73	9.82 ± 6.12	-38.69	<0.001
pNN50 (%)	20.45 ± 6.02	4.93 ± 3.33	-15.52	<0.001	12.76 ± 4.76	2.49 ± 1.80	-10.27	<0.001

Adjusted regression analysis showed that overweight status was independently associated with lower baseline RMSSD, lower post-exercise RMSSD, reduced post-exercise HF power, and lower PFI (all p ≤ 0.001). Mixed-effects modelling demonstrated significant group × time interactions for SDNN, RMSSD, and HF power, whereas mean HR and LF/HF ratio did not show significant interaction effects (Table 4).

**Table 4:** Adjusted regression and mixed-effects analysis of key heart rate variability outcomes CI, confidence interval; RMSSD, root mean square of successive differences; HF, high-frequency power; PFI, Physical Fitness Index; HR, heart rate; SDNN, standard deviation of normal-to-normal intervals; LF/HF, low-frequency to high-frequency power ratio

Analysis	Estimate / statistic	95% CI	p-Value
Baseline RMSSD: overweight effect	β = -20.23 ms	-24.61 to -15.85	<0.001
Post-RMSSD adjusted for baseline	β = -5.40 ms	-8.52 to -2.29	0.0008
Post-HF power adjusted for baseline	β = -82.64 ms²	-108.91 to -56.38	<0.001
PFI: overweight effect	β = -11.36	-14.14 to -8.58	<0.001
Mixed model interaction: Mean HR	F = 3.197	-	0.0753
Mixed model interaction: SDNN	F = 231.787	-	<0.001
Mixed model interaction: RMSSD	F = 72.711	-	<0.001
Mixed model interaction: HF power	F = 115.513	-	<0.001
Mixed model interaction: LF/HF ratio	F = 1.033	-	0.3106

Secondary analyses showed smaller relative reductions in RMSSD and SDNN in overweight participants compared with the normal BMI group, while the percentage change in HF power was not significantly different. Log-transformed analyses confirmed persistently lower vagal-related HRV in overweight participants. Additionally, waist circumference and WHR showed significant inverse associations with baseline RMSSD and HF power, supporting the relationship between central adiposity and reduced autonomic function (Table 5).

**Table 5:** Secondary and sensitivity analyses of autonomic response and adiposity markers CI, confidence interval; RMSSD, root mean square of successive differences; SDNN, standard deviation of normal-to-normal intervals; HF, high-frequency power; HR, heart rate; (ln)RMSSD, natural logarithm-transformed RMSSD; (ln)HF, natural logarithm-transformed HF power; WHR, waist-to-hip ratio; β, regression coefficient

Analysis	Normal	Overweight	Effect / coefficient	95% CI	p-Value
Percent change RMSSD (%)	-61.88 ± 22.61	-52.97 ± 25.52	Mean difference = -8.91	-15.63 to -2.19	0.010
Percent change SDNN (%)	-65.49 ± 13.62	-51.57 ± 25.24	Mean difference = -13.92	-19.58 to -8.27	<0.001
Percent change HF power (%)	-77.53 ± 14.23	-72.73 ± 23.48	Mean difference = -4.80	-10.21 to 0.62	0.082
Percent change mean HR (%)	73.28 ± 28.65	56.41 ± 27.00	Mean difference = +16.87	9.11 to 24.64	<0.001
(ln)RMSSD baseline: overweight effect	-	-	β = -0.548	-0.664 to -0.433	<0.001
(ln)RMSSD post adjusted: overweight effect	-	-	β = -0.384	-0.629 to -0.138	0.002
(ln)HF power post adjusted: overweight effect	-	-	β = -0.755	-0.984 to -0.526	<0.001
Mixed model interaction: (ln)RMSSD	-	-	F = 7.887	-	0.0055
Mixed model interaction: (ln)HF power	-	-	F = 1.040	-	0.309
Baseline RMSSD vs waist circumference	-	-	β = -0.943 per cm	-	<0.001
Baseline HF power vs waist circumference	-	-	β = -13.349 per cm	-	<0.001
Baseline RMSSD vs WHR	-	-	β = -72.61	-	<0.001
Baseline HF power vs WHR	-	-	β = -1005.70	-	0.016

## Discussion

The present study demonstrated that overweight young adults exhibit lower resting HRV and a poorer early post-exercise autonomic profile compared with normal BMI individuals. At baseline, mean RR interval, SDNN, RMSSD, NN50, and pNN50 were significantly lower, while resting HR was higher in the overweight group. Following the Harvard Step Test, both groups showed significant autonomic changes; however, the overweight group continued to demonstrate lower post-exercise RMSSD, SDNN, and HF power, along with a reduced PFI. In adjusted analyses, overweight status remained independently associated with lower baseline RMSSD, lower post-exercise RMSSD, lower post-exercise HF power, and lower PFI. Mixed-effects modelling further revealed significant group × time interactions for SDNN, RMSSD, and HF power, whereas mean HR and the LF/HF ratio did not demonstrate significant interaction effects. Overall, RMSSD and SDNN emerged as the most consistent markers of autonomic alteration in this dataset.

The lower resting vagal-mediated HRV observed in the overweight group is consistent with findings from previous studies in young and otherwise healthy populations. Surana Gandhi et al. [18] reported reduced HRV across overweight individuals with adverse associations with anthropometric indices, while Soumya et al. [19] similarly demonstrated lower HRV in obese young adults compared with healthy controls. Páramo-Lira et al. [20] further reported that increased adiposity in clinically healthy young adults is associated with altered autonomic cardiorespiratory modulation, supporting the presence of early autonomic impairment prior to overt disease. Collectively, these findings reinforce the concept that excess adiposity in young adults is associated with reduced cardiac vagal modulation at rest.

Post-exercise findings in the present study are particularly relevant. Overweight participants demonstrated persistently lower post-exercise RMSSD and HF power even after adjustment, and significant group × time interactions for RMSSD, SDNN, and HF power suggest that autonomic recovery dynamics differed by adiposity status. These findings are consistent with those of Osailan et al. [21], who reported impaired parasympathetic reactivation after exercise in young obese men, and El Agaty et al. [10], who observed delayed vagal recovery in overweight and obese young adult females. Additionally, a meta-regression by Chiang et al. [22] demonstrated that autonomic alterations persist during exercise and up to 1 hour into recovery, highlighting the importance of early recovery assessment rather than resting values alone. Together, these results indicate that excess adiposity is associated not only with reduced baseline HRV but also with attenuated early autonomic recovery following acute exercise.

An interesting observation in this study was that the percentage reduction in RMSSD and SDNN was greater in the normal BMI group despite lower absolute HRV values in the overweight group at both baseline and post-exercise stages. This does not indicate a superior autonomic response in the overweight group. A more plausible explanation is a reduced autonomic reserve or floor effect, as the overweight group already exhibited suppressed vagal activity at baseline, limiting the magnitude of further decline. This interpretation is supported by sensitivity analysis using log-transformed variables, where ln(RMSSD) remained significantly associated with overweight status and retained a significant group × time interaction, whereas HF power lost statistical significance. These findings suggest that RMSSD is a more stable marker of vagal modulation in this context. This is consistent with prior evidence supporting RMSSD as a reliable short-term indicator of parasympathetic activity under controlled recording conditions [23,24].

Central adiposity emerged as an additional important determinant of autonomic function. In secondary analyses, waist circumference showed a significant inverse association with baseline RMSSD and HF power, with stronger and more consistent effects than WHR. This finding has clinical relevance, suggesting that anthropometric indices may differ in their ability to reflect autonomic dysfunction. Banerjee et al. [25] reported that central obesity influences HRV independently of physical activity in young adults, while Surana Gandhi et al. [18] similarly demonstrated associations between waist-based measures and altered HRV. Páramo-Lira et al. [20] further supported the relationship between body composition and autonomic modulation in healthy young individuals. These findings collectively suggest that central adiposity may be a more sensitive indicator of early autonomic dysregulation than BMI alone.

The present study also has practical implications. A simple step-test-based protocol combined with short-duration ECG recordings was sufficient to demonstrate significant differences in autonomic function between BMI groups. This approach is particularly valuable in resource-limited academic settings where advanced cardiometabolic assessments may not be feasible. Furthermore, the findings support the possibility that early autonomic dysfunction associated with excess adiposity may still be modifiable. A systematic review by Mattos et al. [26] demonstrated that weight loss interventions improve HRV in overweight and obese individuals. At the same time, Sinha et al. [27] reported that physical activity has a generally favorable effect on HRV in this population. Therefore, the reduced HRV observed in overweight participants should be interpreted as an early and potentially reversible physiological alteration rather than a permanent dysfunction.

Limitations

This study has several limitations. First, the study population consisted exclusively of medical students from a single institution, which may limit the generalizability of the findings to broader populations and other age groups. Second, the cross-sectional observational design precludes causal inference and does not permit assessment of whether the observed autonomic alterations translate into future cardiovascular risk. Third, HRV analysis was confined to the first 5 minutes of post-exercise recovery and therefore reflects early autonomic recovery rather than complete recovery dynamics. Fourth, although participants with regular exercise habits, smoking history, alcohol use, and major medical illnesses were excluded, several factors known to influence HRV, including sleep quality, caffeine consumption, psychological stress, and habitual physical activity levels, were not formally quantified; therefore, residual confounding cannot be completely excluded. Fifth, classification of participants was based on BMI rather than direct measures of body composition, and some degree of adiposity misclassification may therefore have occurred.

Additionally, a formal a priori sample size calculation was not performed, which may limit assessment of statistical power. The substantial height difference observed between the study groups may also represent a potential source of residual confounding despite adjustment in multivariable analyses.

Furthermore, detailed device-specific signal acquisition parameters, artifact correction procedures, respiratory monitoring data, and proprietary processing algorithms of the recording system were not available for retrospective verification, which may affect methodological reproducibility. Finally, although HF power demonstrated significant differences in the primary analyses, its interaction effect was attenuated after logarithmic transformation, whereas RMSSD and SDNN remained consistently associated with overweight status across multiple analytical approaches. Despite these limitations, the consistency of findings across baseline comparisons, adjusted regression models, mixed-effects analyses, sensitivity analyses, and central adiposity assessments supports the overall conclusion that overweight young adults exhibit reduced vagal-mediated HRV and a less favorable early post-exercise autonomic recovery profile compared with their normal BMI counterparts.

## Conclusions

Overweight young adults exhibited lower resting HRV and a less favorable early post-exercise autonomic profile compared with normal BMI individuals. Vagal-mediated HRV indices, particularly RMSSD and SDNN, were consistently reduced in the overweight group at both baseline and post-exercise assessment. Waist circumference also demonstrated a significant inverse association with baseline vagal-related HRV measures. These findings suggest that excess adiposity in young adults is associated with alterations in autonomic regulation, as reflected by reduced vagal-mediated HRV indices. Longitudinal studies are required to determine the clinical significance and prognostic implications of these findings.
